# Safety and efficacy of T-cell-redirecting bispecific antibodies for patients with multiple myeloma: a systematic review and meta-analysis

**DOI:** 10.1186/s12935-023-03045-y

**Published:** 2023-09-05

**Authors:** Maryam Noori, Niloufar Yazdanpanah, Nima Rezaei

**Affiliations:** 1 Student Research Committee, School of Medicine, Iran University of Medical Sciences, Tehran, Iran; 2 Research Center for Immunodeficiencies, Children’s Medical Center Hospital, Tehran University of Medical Sciences, Dr. Qarib St, Keshavarz Blvd, 14194 Tehran, Iran; 3 Network of Immunity in Infection, Malignancy and Autoimmunity (NIIMA), Universal Scientific Education and Research Network (USERN), Tehran, Iran; 4 Student Research Committee, School of Medicine, Tehran University of Medical Sciences, Tehran, Iran; 5 Department of Immunology, School of Medicine, Tehran University of Medical Sciences, Tehran, Iran

**Keywords:** Multiple myeloma, BsAbs, Bispecific antibodies, Relapse, Hematological, anemia, Neutropenia

## Abstract

**Background:**

In recent years, several bispecific antibodies (BsAbs) have been introduced that revolutionized the treatment approach for patients with multiple myeloma (MM). In the present study, we sought for conducting a systematic review and meta-analysis with the aim of evaluating the safety and efficacy of BsAbs in MM patients.

**Methods:**

PubMed, Scopus, Web of Science, and Embase databases were systematically searched on June 10, 2022. Two steps of title/abstract and full-text screening were performed for selecting the relevant articles. The primary endpoint was considered to evaluate the safety of BsAbs by examining the rate of hematologic and non-hematologic adverse effects (AEs). The secondary outcome was set at the efficacy of BsAbs through pooling objective response rate (ORR), (stringent) complete response (sCR/CR), very good partial response (VGPR), and partial response (PR).

**Results:**

Eleven publications with a total of nine evaluable BsAbs were included for qualitative and quantitative data synthesis. Hematologic AEs were more common among patients than non-hematologic events, with the most frequent events being anemia (41.4%), neutropenia (36.4%), and thrombocytopenia (26.3%). The most common non-hematological AE was infection, which occurred in 39.9% of patients, followed by dysgeusia (28.3%), fatigue (26.5%), and diarrhea (25.8%). Besides, 8.1% of patients experienced immune effector cell-associated neurotoxicity syndrome and neurotoxicity occurred in 5.1% of them. Moreover, 59.8% of patients experienced cytokine release syndrome. The pooled rate of deaths attributable to BsAbs was estimated at 0.1%. In terms of efficacy measures, the ORR was achieved in 62.6% of MM patients, and the pooled rates of sCR/CR, VGPR, and PR were 22.7%, 23.0%, and 12.1%, respectively.

**Conclusions:**

In an era with several emerging promising treatments for MM, BsAbs have achieved a high ORR and tolerable AEs in heavily pretreated patients. However, there is still room for developing BsAbs with a lower rate of AEs and capable of bypassing tumor evasion mechanisms.

**Supplementary Information:**

The online version contains supplementary material available at 10.1186/s12935-023-03045-y.

## Introduction

Multiple myeloma (MM) is the second most frequent hematological malignancy around the world [1]. The disease is characterized by the overproduction of plasma cells and subsequent secretion of a high volume of monoclonal immunoglobulins into the blood and urine [2, 3]. In recent years, immunomodulatory imide drugs (IMiDs), proteasome inhibitors (PIs), and monoclonal antibodies (mAbs) have prolonged the overall survival of patients suffering from MM. However, after a period of receiving these agents, most patients become refractory or intolerant to standard treatments. Moreover, despite several treatment options, MM remains incurable with relapses as inevitable parts of the disease course [4, 5]. Therefore, there is an unmet need for revolutionizing conventional therapies and redirecting novel treatments toward validated targets.

Bispecific antibodies (BsAbs) offer a potential immunotherapeutic approach that has been accompanied by promising results in preclinical studies for treating multiple cancers, particularly hematological malignancies such as acute myeloid leukemia, B-cell non-Hodgkin lymphoma, and precursor B-cell acute lymphoblastic leukemia [6]. The mechanism by which the BsAbs facilitate tumor eradication is the engagement of immune cells to a specific receptor of malignant cells, resulting in subsequent activation of the immune cells and tumor lysis. By applying this approach, T cells would be activated independent of antigen presentation on major histocompatibility complex (MHC) molecules; thus bypassing the usual mechanism of tumor cell recognition [7, 8]. Through T cell activation, perforins and granzymes will be released, resulting in T cell-dependent killing of the tumor cell. T cell-redirecting antibodies can be categorized into two main types: full-length IgG-like antibodies and single-chain variable fragment-based antibodies without an Fc domain. IgG-like bispecific antibodies have a longer elimination half-life compared to scFv-based antibodies, allowing for intermittent administration due to their ability to bind to the neonatal Fc receptor [9].

Numerous BsAbs have been developed in recent years for improving the survival of MM patients. Herein, we sought for conducting a systematic review and meta-analysis with the aim of evaluating the safety and efficacy of BsAbs in MM patients.

## Methods

We have prepared the present study according to the Preferred Reporting Items for Systematic Reviews and Meta-Analyses (PRISMA) statement [10]. The protocol was submitted to PROSPERO database with the registration number CRD42022353357.

### Search strategy

We conducted a systematic review and meta-analyses focused on the efficacy and safety of BsAbs targeting T cells and plasma cells in relapsed/refractory multiple myeloma (RRMM) patients. PubMed, Scopus, Web of Science, and Embase databases were systematically searched on June 10, 2022, using search terms, including “bispecific antibodies”, “dual-targeted antibodies”, “BiTE”, “multiple myeloma”, and other relevant or equivalent terms. No filter was applied to any field of the search. Detailed information for the search strategy is provided in Additional file 1: Table S1. Additional relevant records were retrieved by searching the meeting libraries, including the American Society of Hematology (ASH) and the American Society of Clinical Oncology (ASCO) to identify eligible published conference abstracts. The title and abstract of the recorded publications were screened and the full-texts of the selected articles were assessed for qualification by two independent reviewers; any disagreements were discussed and finally referred to a third reviewer for resolution. The primary endpoint was considered evaluating the safety of BsAbs via examining the rate of hematologic and non-hematologic adverse effects (AEs) following the administration of BsAbs, particularly cytokine release syndrome (CRS), immune effector cell-associated neurotoxicity syndrome (ICANS), neurotoxicity, and death. The secondary outcome was considered assessing the efficacy of BsAbs through pooling objective response rate (ORR), (stringent) complete response (sCR/CR), very good partial response (VGPR), and partial response (PR).

### Eligibility criteria

The studies were included if [1]: they were designed as a single-arm clinical trial [2], participants were diagnosed with RRMM according to the criteria of the International Myeloma Working Group [3], at least one group received monotherapy dosing of bispecific antibodies targeting T cells and malignant plasma cells, and [4] the efficacy and safety endpoints were reported.

The exclusion criteria were as follows [1]: reviews, commentaries, in vitro studies, and studies conducted on mice and non-human primates [2], studies that evaluate the effect of dual-targeting CAR-T cell therapies, and [3] studies that enrolled patients with the diagnosis of hematologic malignancies other than multiple myeloma.

### Data extraction

Two investigators independently reviewed the title and/or abstract to identify the potentially eligible studies. The full-texts of the potentially eligible studies were retrieved and independently reviewed for eligibility by two reviewers. Any conflict between the two reviewers was resolved by a third investigator. We used a pre-piloted excel sheet for data extraction. The following information was extracted: [1] bibliographic data including the first author name, year of publication, country of origin, and clinical trial identifier number [2], demographic information and characteristics of the participants including the baseline number of participants, age, sex, the rate of high-risk cytogenetics, the rate of triple refractory patients, and the number of prior lines of therapy [3], details of the interventions including the dose, schedule, and target of BsAbs [4], study methodology including duration, recruitment, inclusion and exclusion criteria, and [5] information for the assessment of the risk of bias.

In addition, the data related to outcome measures such as the number of patients who experienced sCR/CR, VGPR, PR, undetectable minimal residual disease (MRD), as well as median duration of response (DOR), and median time to any response, as well as the number of all hematologic and non-hematologic AEs and grade ≥ 3 AEs were also collected.

### Quality assessment

The Methodological Index for Non-randomized Studies (MINORS) was used to assess the methodological quality of the included studies [11]. The MINORS scale contains eight items for non-comparative studies, including study aims, consecutive patient inclusion criteria, prospective pooling of data, endpoint consistent with the study aim, unbiased evaluation of endpoints, follow-up period, loss to follow-up less than 5%, and prospective calculation of the study size. Each item were scored 0 (not reported), 1 (reported but inadequate), or 2 (reported and adequate) [11].

### Data analysis

We were able to identify 11 publications with nine different specific antibodies. Overlapping publications were recognized for several studies. In such a situation, we selected the most recent phase I dose escalation study for safety assessment and trials reporting the effectiveness of a recommended phase II dose (RP2D) or a certain dose with the highest effectivity were considered for efficacy analysis. Consequently, seven publications were identified for both safety and efficacy assessment [12–18], while for two trials that have further assessed the safety and efficacy of a RP2D, we considered the dose escalation publications for safety analysis [19, 20] and the publications evaluating the effectiveness of RP2D for efficacy analysis [21, 22].

We conducted a meta-analysis for proportions to estimate the overall proportion of AEs, ORR, sCR/CR, VGPR, and PR. Heterogeneity was judged based on *I*^*2*^ statistics. A fixed effect model was used if the between-study heterogeneity test statistics was *I*^*2*^ ≤ 50%, otherwise, a random effect model was utilized [23]. The results were reported as proportions with a 95% confidence interval (CI). Besides, whenever the analysis was not feasible due to between-study variation and data scarcity, we summarized the information qualitatively. All statistical analyses were carried out using STATA software version 17.

## Results

**Fig. 1:**
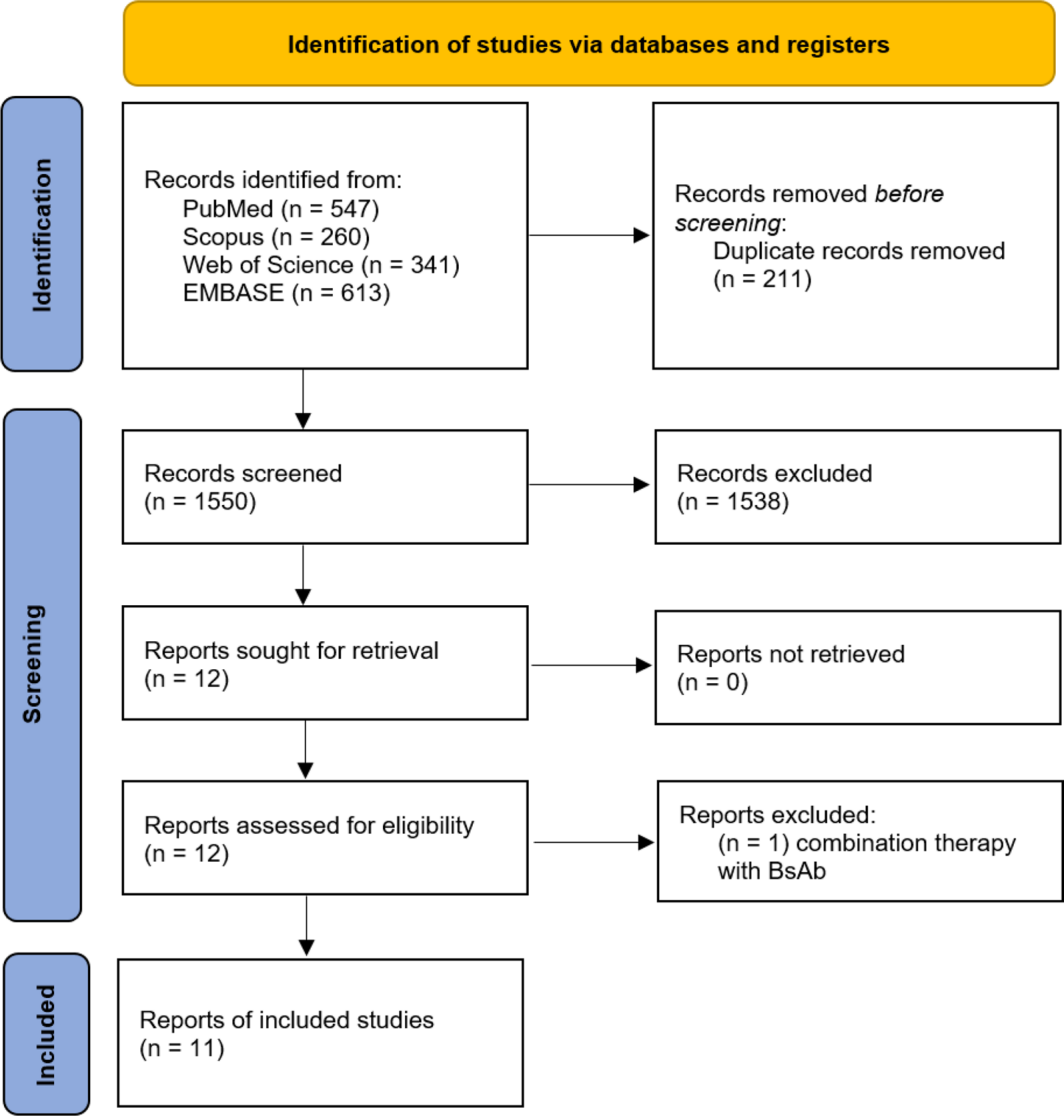
Flow diagram of study selection process

A total of 1,761 records were identified through the initial search of PubMed (n = 547), Scopus (n = 260), Web of Science (n = 341), and Embase (n = 613) databases. After the removal of 211 duplicated records, 1550 publications underwent title/abstract screening, leaving 12 papers to be evaluated by full-texts. At this step, one study was excluded due to the administration of a BsAb as combination therapy [24]. Consequently, 11 potential records were sought for qualitative and quantitative data synthesis. Of these, eight articles [12–15, 17–19, 21] were conference abstracts and three [16, 20, 22] were published in peer-reviewed journals. The PRISMA flow diagram of study selection is depicted in Fig. 1.

### Study quality

The quality assessment of the included studies is presented in Additional file 1: Table S2. Briefly, all included trials (n = 9) provided adequate information on a clearly stated aim, prospective collection of data, loss to follow-up of less than 5%, and endpoints appropriate to the aim of the study. Six studies provided adequate information on the follow-up period appropriate to the aim of the study and two studies had adequate information on the inclusion of consecutive patients. Furthermore, none of the studies provided information on the reported prospective calculation of the study size and unbiased assessment of the study endpoint. Overall, all trials scored between 10 and 12 points, with a mean score of 10.9.

### Study characteristics

Eleven publications with a total of nine evaluable BsAbs were included. For seven BsAbs, the plasma-cell recognition target was BCMA [12–16, 18, 20], while Fc receptor-like protein 5 (FcRH5) was targeted for one of the BsAbs [17, 22], and G Protein-Coupled Receptor Class C Group 5 Member D (GPRC5D) for the other one [19, 21]. The median age of the 853 included patients ranged from 63 to 68 comprising 473 (55.4%) male patients. Among 811 evaluable patients, 578 (71.3%) were refractory to triple classes of routine MM medications (i.e., PI, IMID, and anti-CD38 monoclonal antibody) and the median prior line of therapy ranged from four to eight. Furthermore, 29.1% (165/568) of all patients had high-risk cytogenetics including 17p deletion, t(14;16), and t(14;20). The detailed characteristics of included trials are summarized in Table 1. In addition, Additional file 1: Table S3 provided descriptions regarding the inclusion and exclusion criteria of participants for each trial, the percentage of patients in each eastern cooperative oncology group (ECOG) performance status and international staging system (ISS) stage, and the prior history of autologous or allogenic stem-cell transplantation.

**Table 1:** Baseline characteristics of BsAbs and demographic information of included patients

Bispecific antibody	NCT Identifier	Sponsor	Type of BsAb	Target	Route	Dose	Schedule	Number of patients	Age (Year)	Sex (No. of males) (%)	High risk cytogenetics^*^ (%)	Refractory to triple classes^**^ (%)	No. of prior lines of therapy
AMG 420 (BI 836909) (16)	NCT02514239	Amgen	Short half-life BiTE	BCMA.CD3	IV	0.2–800 µg/d	AMG 420 given for up to 10 cycles, with each 6-week cycle including 4 weeks of continuous intravenous administration followed by 2 weeks off treatment.	42	Median (range) 65 (39–79)	27 (64.3)	14/42 (33.3)	N/A	4 (2–13)
AMG 701 (13)	NCT03287908	Amgen	Extended half-life BiTE	BCMA.CD3	IV	0.015-18 mg	Weekly IV infusion for 4 weeks treatment cycles.	85	Median (range) 64 (34–83)	44 (51.8)	N/A	53 (62.4)	6 (2–25)
Cevostamab (BFCR4350A/RO7187797) (17)	NCT03275103	Genetech	IgG1-based Fc region	FcRH5.CD3	IV	0.05–160 mg	In the single-set up cohorts, the step dose (0.05-3.6 mg) was given on C1 Day (D) 1 and the target dose (0.15-198 mg) on C1D8. In the double set up cohorts, the step doses were given on C1D1 (0.3-1.2 mg) and C1D8 (3.6 mg), and the target dose (60-160 mg) on C1D15.	161	Median (range) 64 (33–82)	94 (58.4)	67/95 (70.5)	136 (84.5)	6 (2–18)
CC-93269 (12)	NCT03486067	Bristol Myers Squibb	IgG1-based Fc region	BCMA.CD3	IV	0.15-10 mg	CC-93,269 was administered over 2 h on days 1, 8, 15, and 22 of cycles 1 to 3, on days 1 and 15 of cycles 4 to 6, and on day 1 of cycle 7 onwards, all in 28-day cycles.	30	Median (range) 64 (42–78)	21 (70.0)	9/30 (30.0)	20 (66.7)	5 (3–13)
REGN5458 (18)	NCT03309111	Regeneron	VelociBi™ Fc region	BCMA.CD3	IV	3-800 mg	16 weekly infusions of REGN5458, followed by every two-week dosing.	73	Median (range) 64 (41–81)	34 (46.6)	10/57 (17.5)	14 (19.2)	5 (2–17)
Elranatamab (PF-06863135) (15)	NCT03269136	Pfizer	IgG2a Fc region	BCMA.CD3	SC	80-1000 µg/kg	Patients received Elranatamab at 80, 130, 215, 360, 600, and 1000 µg/kg SC weekly.	30	Median (range) 63 (46–80)	13 (43.3)	7/30 (23.3)	26 (86.7)	8 (3–15)
TNB-383B (ABBV-383) (14)	NCT03933735	TeneoBio	IgG4 Fc region	BCMA.CD3	IV	0.25–120 mg	TNB-383B was administered IV over 1–2 h every 3 weeks.	118	Median (range) 68 (35–88)	66 (55.9)	N/A	72 (61.0)	5 (1–15)
Teclistamab (JNJ-64007957) (20, 22)	NCT03145181	Janssen	IgG4 Fc region	BCMA.CD3	SC/IV	IV: 0.3–720 µg/kg SC: 80-3000 µg/kg	0·3 µg/kg, administered intravenously on days 1 and 15 of 28-day cycles. The schedule was changed to once per week intravenous dosing (days 1, 8, and 15 of 21-day cycles) based on emerging pharmacokinetic data. Once per week subcutaneous dosing of teclistamab was also tested.	157	Median (IQR) 63 (57–69)	85 (54.1)	39/157 (24.8)	128 (81.5)	6 (IQR: 4–7)
Talquetamab (JNJ-64,407,564) (19, 21)	NCT03399799	Janssen	IgG4 Fc region	GPRC5D.CD3	SC/IV	IV: 0.5–180 µg/kg SC: 5-800 µg/kg	Set-up dose at week 1 followed by a full dose administrated weekly or biweekly for cycle 1 and beyond.	157	Median (range) 64 (33–80)	89 (56.7)	20/157 (12.7)	129 2.2)	6 (2–20)

* 17p deletion, t(14;16), t(14;20), ** PI, IMID, and anti-CD38 monoclonal antibody. Abbreviations: FcRH5: Fc receptor-homolog 5; BCMA: B-cell maturation antigen; GPRC5D: G-protein coupled receptor family C group 5 member D; IgG: Immunoglobulin G; SC: Subcutaneous; IV: Intravenous; N/A: Not available

### Safety analysis

**Fig. 2:**
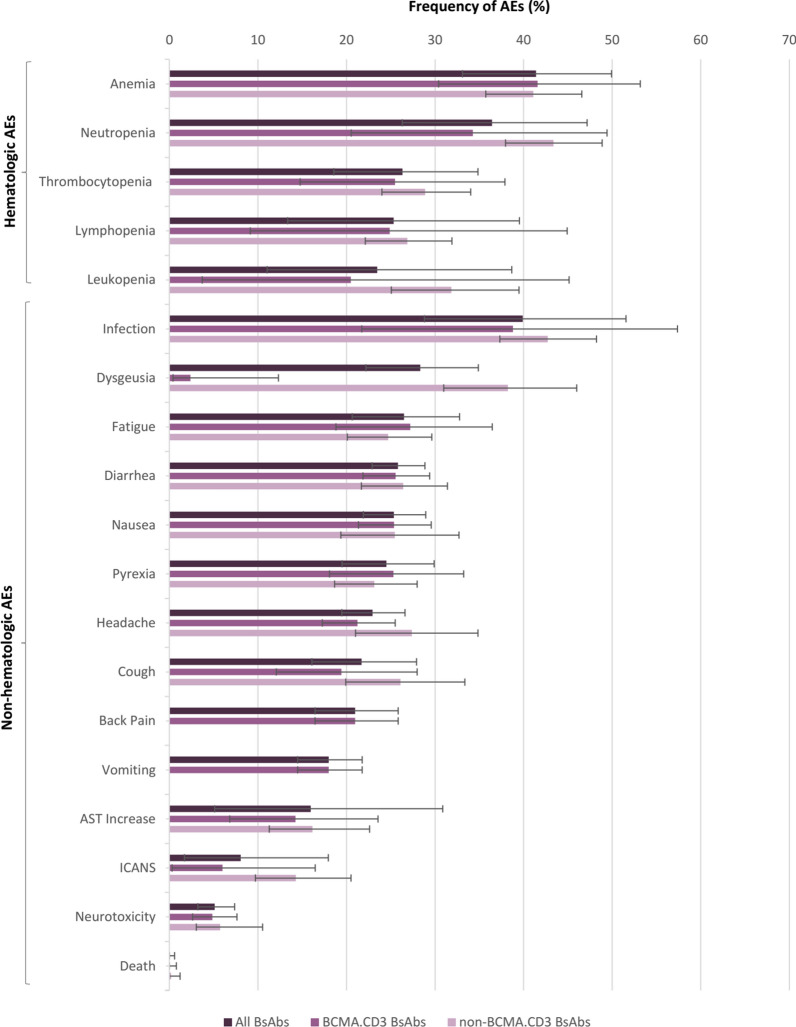
All hematologic and non-hematologic adverse events following administration of bispecific antibodies for patients with multiple myeloma

 Among 853 safety evaluable patients, hematologic AEs were more common among patients, with the most frequent event being anemia (41.4%, 95% CI 33.1–49.9) followed by neutropenia (36.4%, 95% CI 26.3–47.2), thrombocytopenia (26.3%, 95% CI 18.6–34.8), lymphopenia (25.3%, 95% CI 13.3–39.5), and leukopenia (23.5%, 95% CI 11.0–38.7) (Additional file 1: Fig. S1). The most common non-hematological AE was infection, which occurred in 39.9% (95% CI 28.8–51.6) of patients, and then dysgeusia (28.3%, 95% CI 22.2–34.9), fatigue (26.5%, 95% CI 20.6–32.8), diarrhea (25.8%, 95% CI 22.9–28.9), nausea (25.3%, 21.9–28.9), pyrexia (24.5%, 95% CI 19.5–29.9), headache (22.9%, 95% CI 19.5–26.6), cough (21.7%, 95% CI 16.1–27.9), back pain (21.0%, 95% CI 16.5–25.8), vomiting (18.0%, 95% CI 14.5–21.8), and aspartate aminotransferase (AST) rise (16.0%, 95% CI 5.1–30.9) (Additional file 1: Fig. S2). Except for anemia, fatigue, and pyrexia, all AEs were more frequent among patients receiving non-BCMA.CD3 targeted BsAbs than those who received BCMA.CD3 targeted BsAbs. Besides, 8.1% (95% CI 1.7–18.0) of patients experienced ICANS and neurotoxicity occurred in 5.1% (95% CI 3.2–7.4) of them. The pooled rate of deaths attributable to BsAbs was 0.1% (95% CI 0.0–0.6) occurring in three patients receiving CC–93,269, Cevostomab, and Teclistamab (Fig. 2).

**Fig. 3:**
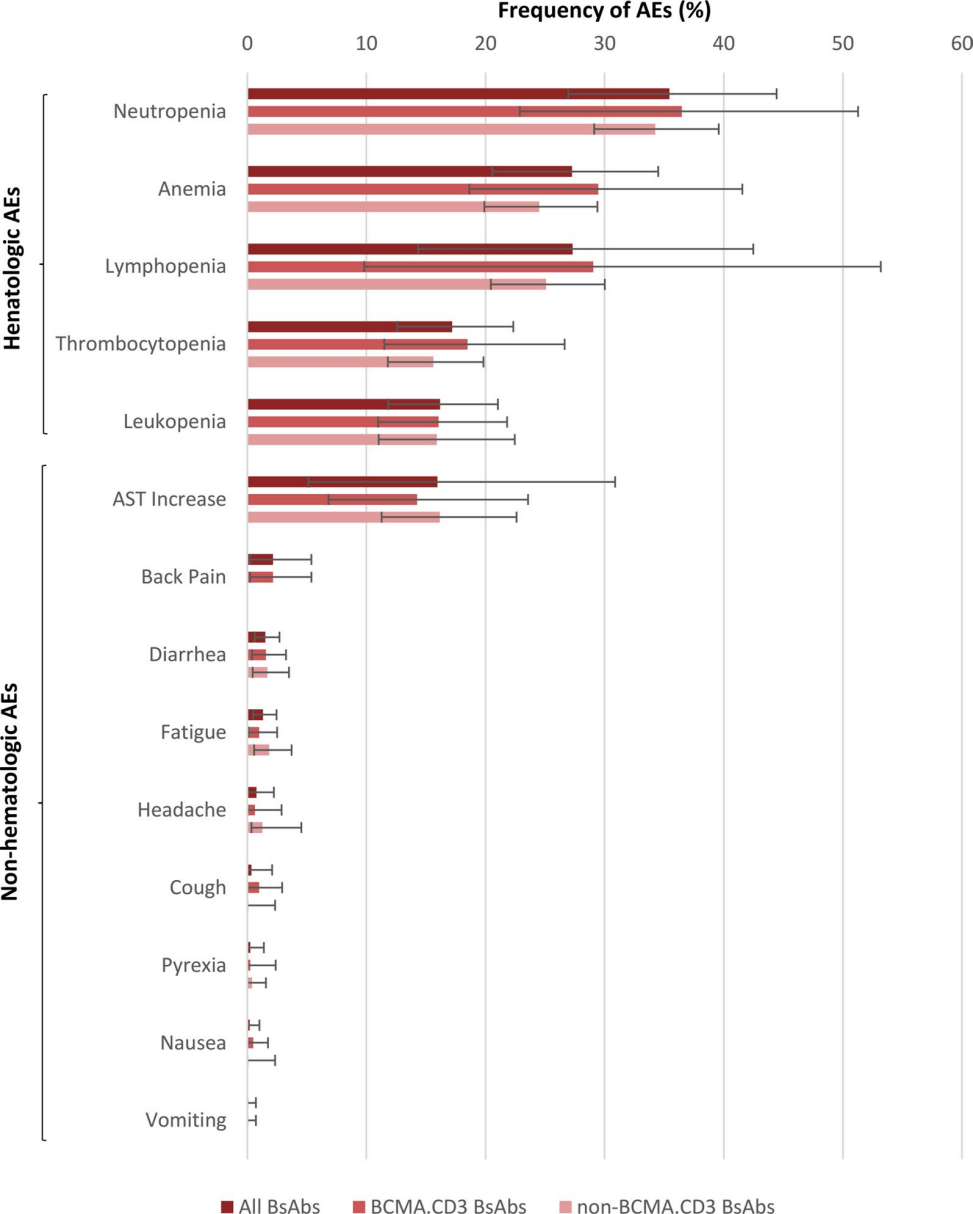
Grade ≥3 hematologic and non-hematologic adverse events following administration of bispecific antibodies for patients with multiple myeloma

In terms of hematologic grade ≥ 3 AEs, neutropenia with a frequency of 35.4% (95% CI 26.9–44.4) was the most common AE, followed by anemia (27.3%, 95% CI 20.6–34.5), lymphopenia (27.3%, 95% CI 14.4–42.5), thrombocytopenia (17.2%, 95% CI 12.6–22.3), and leukopenia (16.2%, 95% CI 11.8–21.1) (Additional file 1: Fig. S3). Among non-hematologic grade ≥ 3 AEs, AST increase had the highest frequency by 16.0% (95% CI 5.1–30.9), followed by back pain (2.1%, 95% CI 0.2–5.4), diarrhea (1.5%, 95% CI 0.6–2.7), fatigue (1.3%, 95% CI 0.5–2.5), headache (0.8%, 95% CI 0.0–2.2), cough (0.3%, 95% CI 0.0–2.1), pyrexia (0.3%, 95% CI 0.0–1.4), nausea (0.2%, 95% CI 0.0–1.0), and vomiting (0.0%, 95% CI 0.0–0.7) (Additional file 1: Fig. S4). Furthermore, while the rate of all hematologic AEs was higher for patients who received BCMA targeted BsAbs, the rate of non-hematologic AEs except for cough and nausea was higher in non-BCMA targeted BsAb group (Fig. 3).

**Fig. 4:**
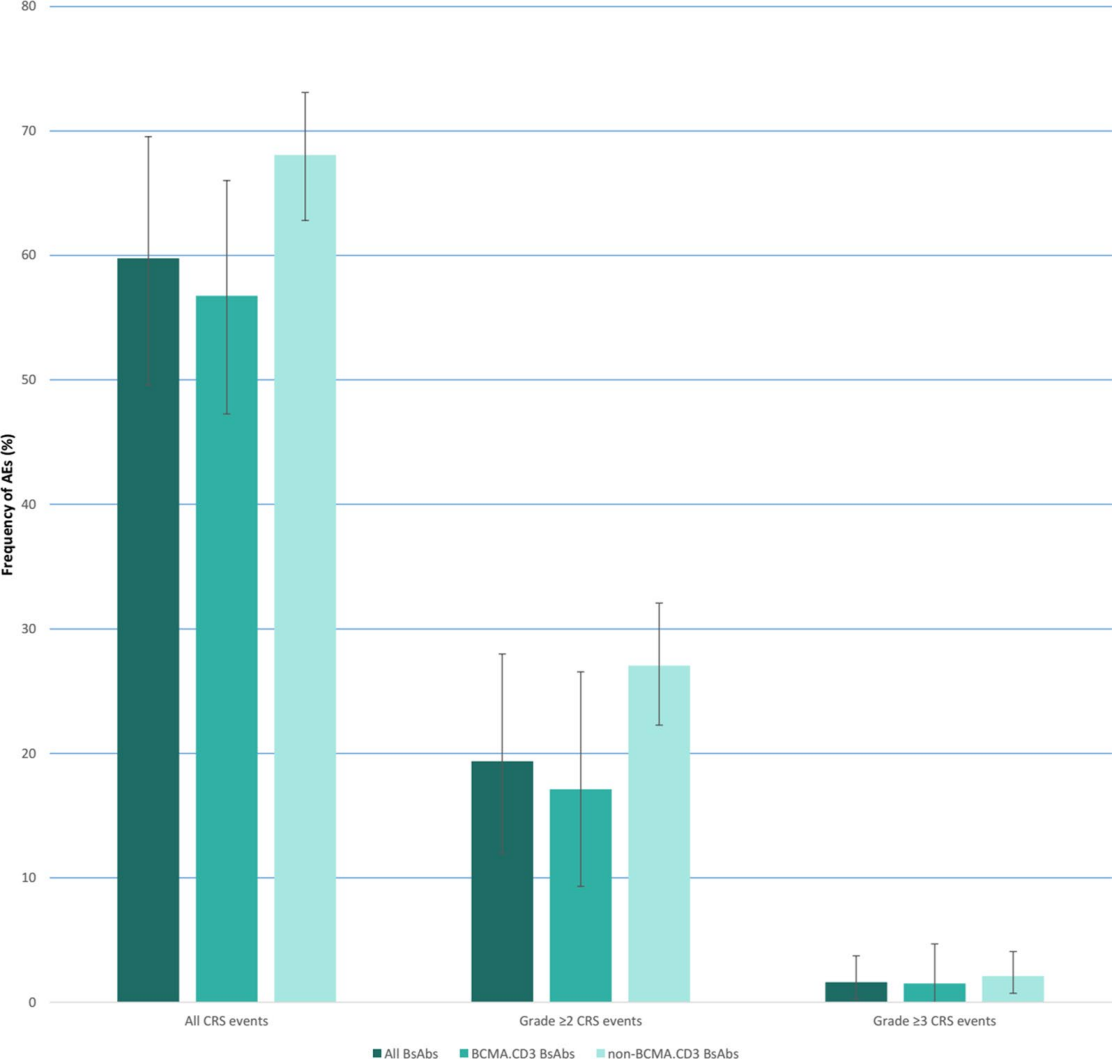
Cytokine release syndrome (CRS) events following administration of bispecific antibodies for patients with multiple myeloma

Moreover, 59.8% (95% CI 49.6–69.5) of patients experienced CRS, which varied greatly from 38.1% for AMG 420 to 80.7% for Cevostamab (Additional file 1: Fig. S5). The pooled CRS rate in studies using BCMA.CD3 structure (56.7%, 95% CI 47.2–66.0%) was lower than studies targeting myeloma cells through receptors other than BCMA (68.1%, 95% CI 62.8–73.1%). Severe CRS, defined as the CRS events of grade 3 or higher was evident in 1.6% (95% CI 0.3–3.7) of cases. In addition, the rate of CRS grade ≥ 2 events was 19.4% (95% CI 12.0–28.0) among participants (Fig. 4). The median time to CRS onset after administration of BsAbs was between 10 h to 2 days and the median duration of CRS ranged from 15 h to three days. The CRS events were resolved mostly through the administration of tocilizumab and corticosteroids.

### Efficacy analysis

**Fig. 5:**
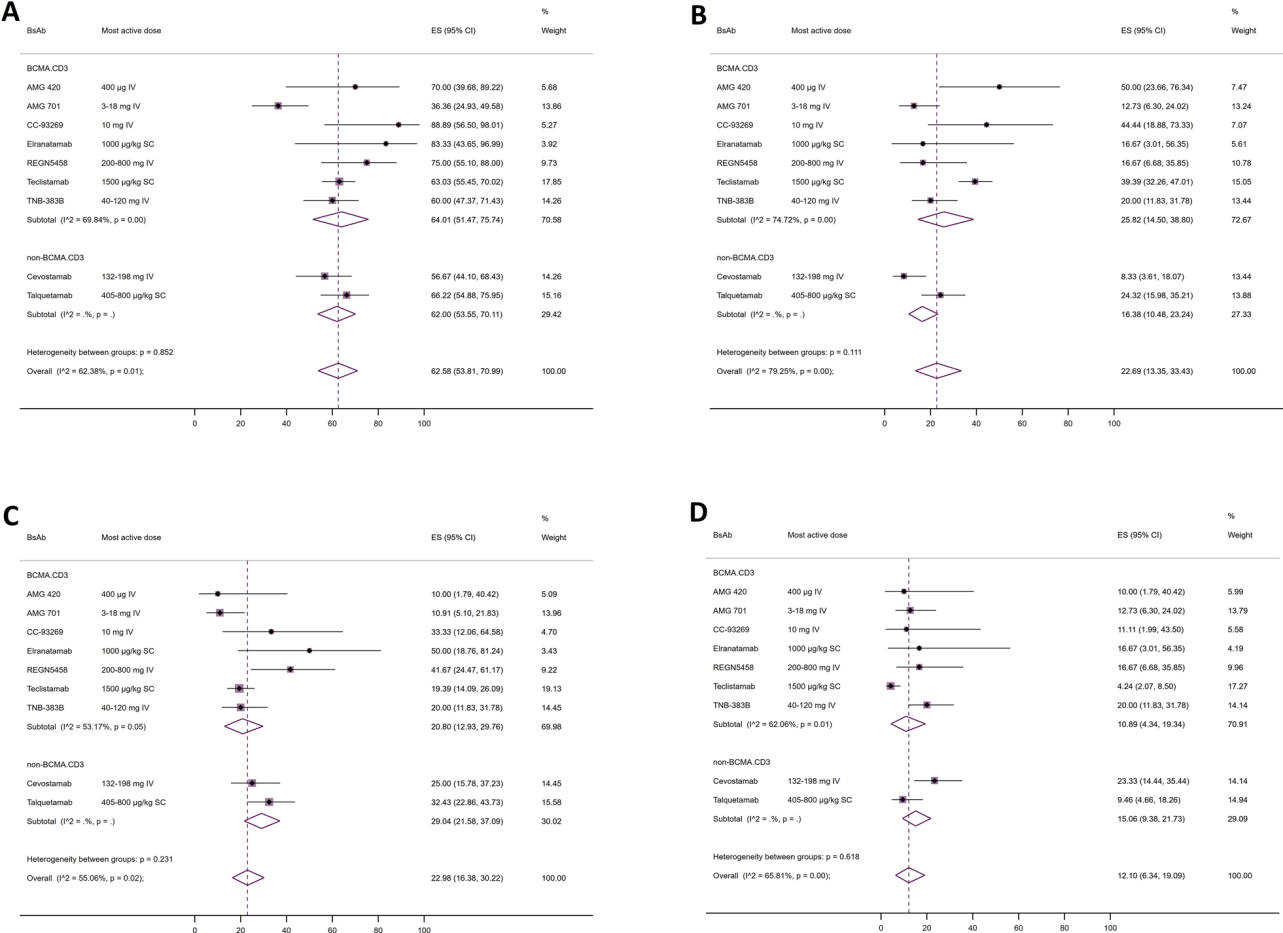
Efficacy of bispecific antibodies for patients with multiple myeloma. **A** Objective response rate, **B** (Stringent) complete response, **C** Very good partial response, and **D** Partial response

A total of 463 RRMM patients were evaluable for clinical response. The overall ORR was 62.6% (95% CI 53.8–71.0), which was slightly higher in studies targeting BCMA in myeloma cells (64.0%, 95% CI 51.5–75.7) as compared to BsAbs targeting non-BCMA receptors (62.0%, 95% CI 53.6–70.1). Moreover, the pooled rates of sCR/CR, VGPR, and PR were 22.7% (95% CI 13.3–33.4), 23.0% (95% CI 16.4–30.2), and 12.1% (95% CI 6.3–19.1), respectively. While BCMA.CD3 targeted BsAbs represented a higher rate of sCR/CR responses, non-BCMA.CD3 targeted BsAbs showed higher rates of VGPR and PR (Fig. 5). Of the 106 MRD evaluable responding patients, 82 subjects (77.4%) achieved undetectable MRD. Median DOR was not reached in five trials, while it was 8.4 months for AMG 420, 11.5 months for Cevostamab, 18.4 months for Teclistamab, and 10.2 months for Talquetamab trials. Finally, the median time to any response ranged from 22 days to 4.1 weeks (Additional file 1: Table S4).

## Discussion

The present systematic review and meta-analysis is the most comprehensive study aiming at evaluating the safety and efficacy of BsAbs in patients suffering from MM. As of June 2022, the trial results of nine unique BsAb products targeting T cells along with malignant myeloma cells have been published. All BsAbs have been designed to target CD3 molecule on T cells. In addition, the myeloma cell target of seven BsAbs was chosen to be BCMA, while the other two BsAbs targeted GPRC5D and FcRH5. Overall, our pooled analysis demonstrated that BsAb therapy offered promising outcomes with an acceptable safety profile in RRMM patients.

The first idea of using BsAbs dates back to the 1980s when scientists hypothesized redirecting of effector immune cells to tumor cells through antibodies that recognize two different epitopes or antigens [25, 26]. Blinatumomab which targets CD19 on cells of B-lineage origin and CD3 on T cells was the first BsAb approved by the US Food and Drug Administration (FDA) for adult and pediatric patients with B cell precursor acute lymphoblastic leukemia (ALL) [27]. More recently, BsAbs caught attention for the treatment of MM following the promising results of phase 1 clinical trials [28]. Among BsAbs that have been designed for MM patients, only Teclistamab have gained FDA approval so far; however, Teclistamab has been recently recommended by the European Medicine Agency (EMA) for the treatment of RRMM patients [29].

Our results showed that 61.2% and 24.3% of all treated MM patients with BsAbs achieved ORR and sCR/CR, respectively. Besides, we found that there was no obvious difference between the ORR of BsAbs targeting BCMA (60.8%) and those targeting non-BCMA molecules on malignant cells (62%). In a meta-analysis conducted by Yu and colleagues, the prescription of blinatumomab resulted in a pooled CR rate of 45% for ALL patients and 20% for non-Hodgkin lymphoma patients [30]. Furthermore, while we observed 77.4% undetectable MRD among MM patients receiving BsAb, blinatumomab caused 42% of ALL patients to become MRD negative [30]. On the other hand, CAR T cells have shown superior benefits with an ORR of 88% and an MRD negativity rate of 79% among MM patients [31]. MRD status is one of the most useful prognostic factors in MM patients. In comparison with those achieving CR with MRD positive status, MM patients who become MRD negative have longer overall and progression free survival [32, 33]. Despite a high rate of MRD negativity, it remains unclear whether BsAbs have the potential to elicit long-lasting responses in MM patients. Herein, designing trials with longer follow-up durations for patients who reached MRD negativity will be necessary for understanding whole dimensions of responses induced by BsAbs.

We found that AEs such as cytopenia and infections are common after BsAb therapy. Non-transformed B cells and plasma cells may also express the targeted antigen on MM malignant cells; thus, the host normal B cells could be lysed during BsAb therapy as a result of identifying identical antigens on tumor and healthy cells, predisposing the patients to a high risk of depleted immune cells and infection. This is obviously evident in our meta-analysis that infection was the most common non-hematological AE, occurring in 41.3% of all BsAb recipients. Similarly, the risk of infection after blinatumomab therapy ranged from 34 to 44% in patients with hematological malignancies [34]. This increased risk of infection might be resolved for the future BsAbs by improving the specificity through targeting multiple specific antigens on tumor cells [7].

CRS is an acute systemic inflammatory syndrome, which occurs frequently after treatment with immunotherapeutic agents, particularly chimeric antigen receptor (CAR) T cells and BsAbs [35]. Activation of many T cells and other effector immune cells is responsible for releasing a high amount of cytokines. The severity of CRS could be associated with the type of underlying malignancy as well as the type, dose, and schedule of immunotherapy. In our study, the overall rate of CRS events was 60.4% with a higher rate of 68.1% for BsAbs that target BCMA and a lower rate of 55.7% for BsAbs that target receptors other than BCMA on malignant cells. The rate of severe CRS events was generally low and documented in 1.4% of patients. A meta-analysis which was conducted on 23 different CAR T cell products for MM patients has reported the incidence of overall and severe CRS events to be 80.3% and 14.1%, respectively [36], a higher rate than what we reported for BsAbs. In our study, the CRS events were mainly managed through the administration of tocilizumab and corticosteroids. However, a recent investigation has revealed that the anti-tumor function of T cells mediated by BsAbs can be substantially compromised by using corticosteroids, while tocilizumab represented significant CRS attenuation without affecting the efficacy of BsAbs [37].

Several trials have been launched in recent years for investigating novel BsAbs that have been designed to induce a lower rate of AEs and conquer tumor evasion. Improving the functionality of BsAbs could occur in multiple ways. In this matter, the downregulation of the targeted antigens by tumor cells is a well-known mechanism for impairing the efficacy of antibodies. Constructing multivalent BsAbs that enhance target avidity or trispecific antibodies that bind to more than one antigen on malignant cells might be the way to overcome the resistance [38]. Furthermore, multi-target antibodies may also be useful for preventing B cell aplasia, lymphocyte depletion, and subsequent risk of infection by specifying that certain cells expressing a combination of particular antigens are targeted and healthy cells would not be invaded by the antibodies [38]. Besides, the BsAb therapy in MM patients may take advantage of combination with immune checkpoint inhibitors (ICIs) by avoiding T cell exhaustion [39]. The PD-1/PD-L1 signaling pathway is the hallmark of tumor immunosuppression and T cell deactivation. An over-expression of PD-1 and PD-L1 molecules has been noted in MM patients [40]. Moreover, induced T cell exhaustion through high expression of PD-L1 has been reported while using BsAb therapy [41, 42]. In this regard, addition of ICIs that block the PD-1/PD-L1 pathway to BsAbs can counter T cell exhaustion and increase their activation. Moreover, a potential limitation in the function of BsAbs is the activation of independent T cells, which may also activate unnecessary Tregs [27]. Addition of therapeutic Treg depletion to BsAb therapy may be useful in overcoming immunosuppression. To this end, applying methods that fight against the immunosuppressive microenvironment of bone marrow could be crucial in enhancing the effectiveness of BsAb therapy.

To the best of our knowledge, this is the first systematic review and meta-analysis pooling the response rates and rates of AEs following administration of BsAbs for patients with MM. Nevertheless, we acknowledge that our study is subjected to several drawbacks that should be considered when interpreting our findings. Firstly, the trials of BsAbs are mainly in their early phases and none of them has published phase 3 results. Therefore, the lack of a control group among included studies may impact the validity of the conclusions. Secondly, most of the trials were in the dose escalation stage while some of them were conducted based on a certain RP2D of BsAbs. This heterogeneous primary design of the included trials may further increase the uncertainties. Thirdly, the variations in basic parameters of the studies, such as eligibility criteria, the model of BsAbs, and various ranges of efficacious doses and schedules may prevent drawing a generalizable conclusion. Fourthly, we only included trials that assessed the safety and efficacy of T cell redirected BsAbs; thus, the outcomes of BsAbs that target innate immunity could be further evaluated in future systematic reviews and meta-analyses. Lastly, the main part of our data was extracted from the published abstracts of the congress, which have not undergone a full process of peer-reviewing and have not provided detailed information.

### Conclusion

In an era of several emerging promising treatments for MM, BsAbs have achieved a high ORR and tolerable AEs in heavily pretreated patients. However, there is still room for developing BsAbs that induce a lower rate of AEs and are capable of bypassing tumor evasion mechanisms. In addition, the initiation of phase 3 randomized controlled trials with a long follow-up duration, which compare the safety and efficacy of current BsAbs with conventional MM treatments, is highly recommended. Finally, combination therapies provided countless opportunities that can be integrated into the BsAb treatment approach and improve the survival of treated patients.

### Supplementary Information

**Additional file 1: Table S1. **Search strategy. **Table S2.** MINORS scale for quality assessment of included studies. **Table S3. **Baseline characteristics of included studies. **Table S4. **Efficacy parameters for different bispecific antibodies. **Figure S1.** All hematologic adverse events following administration of bispecific antibodies for patients with multiple myeloma. **A** Anemia, **B** Neutropenia, **C** Thrombocytopenia, **D** Leukopenia, and **E** Lymphopenia. **Figure S2.** All non-hematologic adverse events following administration of bispecific antibodies for patients with multiple myeloma. **A** Infection, **B** Dysgeusia, **C** Fatigue, **D** Diarrhea, **E** Nausea, **F** Pyrexia, **G** Headache, **H** Cough, **I** Back pain, **J** Vomiting, **K** AST increase, **L** ICANS, **M** Neurotoxicity, and **N** Death. Abbreviations: AST: Aspartate Transferase; and ICANS: Immune effector cell-associated neurotoxicity syndrome. **Figure S3.** Grade ≥3 hematologic adverse events following administration of bispecific antibodies for patients with multiple myeloma. **A** Neutropenia, **B** Anemia, **C** Lymphopenia, **D** Thrombocytopenia, and **E** Leukopenia. **Figure S4.** Grade ≥3 non-hematologic adverse events following administration of bispecific antibodies for patients with multiple myeloma. **A** AST increase, **B** Back pain, **C** Diarrhea, **D** Fatigue,**E** Headache, **F** Cough, **G** Nausea, **H** Vomiting, and **I** Pyrexia. Abbreviations: AST: Aspartate Transferase. **Figure S5.** ‎Cytokine release syndrome (CRS) events following administration of bispecific antibodies for patients with multiple myeloma. **A** All events, **B** CRS events grade ≥2, and**C** CRS events grade ≥3.

## Data Availability

Not applicable.
