# Supplementary material for: Safety and efficacy of T-cell-redirecting bispecific antibodies for patients with multiple myeloma: a systematic review and meta-analysis
Source: Cancer Cell Int. 2023 Sep 5;23:193. doi: 10.1186/s12935-023-03045-y (PMC10478206; doi:10.1186/s12935-023-03045-y)
Supplement: Supplementary file 1 — Additional file 1: Table S1. Search strategy. Table S2. MINORS scale for quality assessment of included studies. Table S3. Baseline characteristics of included studies. Table S4. Efficacy parameters for different bispecific antibodies. Figure S1. All hematologic adverse events following administration of bispecific antibodies for patients with multiple myeloma. A Anemia, B Neutropenia, C Thrombocytopenia, D Leukopenia, and E Lymphopenia. Figure S2. All non-hematologic adverse events following administration of bispecific antibodies for patients with multiple myeloma. A Infection, B Dysgeusia, C Fatigue, D Diarrhea, E Nausea, F Pyrexia, G Headache, H Cough, I Back pain, J Vomiting, K AST increase, L ICANS, M Neurotoxicity, and N Death. Abbreviations: AST: Aspartate Transferase; and ICANS: Immune effector cell-associated neurotoxicity syndrome. Figure S3. Grade ≥3 hematologic adverse events following administration of bispecific antibodies for patients with multiple myeloma. A Neutropenia, B Anemia, C Lymphopenia, D Thrombocytopenia, and E Leukopenia. Figure S4. Grade ≥3 non-hematologic adverse events following administration of bispecific antibodies for patients with multiple myeloma. A AST increase, B Back pain, C Diarrhea, D Fatigue,E Headache, F Cough, G Nausea, H Vomiting, and I Pyrexia. Abbreviations: AST: Aspartate Transferase. Figure S5. ‎Cytokine release syndrome (CRS) events following administration of bispecific antibodies for patients with multiple myeloma. A All events, B CRS events grade ≥2, andC CRS events grade ≥3. [file 12935_2023_3045_MOESM1_ESM.docx]

**Safety and efficacy of T-cell-redirecting bispecific antibodies for patients with multiple myeloma; A systematic review and meta-analysis**

**Table S1.** Search strategy

|  | Step | Search strategy | No. of results |
| --- | --- | --- | --- |
| PubMed (6.10.2022) | | | |
|  | #1 | “Multiple Myeloma*”[tiab] OR “plasma cell dyscrasia”[tiab] OR “plasma-cell dyscrasia”[tiab] OR “Myelomatosis”[tiab] OR “Myelomatoses”[tiab] OR “Kahler Disease”[tiab] OR “Multiple Myeloma”[MeSH] OR “Myeloma-Multiple*”[tiab] OR “MM”[tiab] | 270,721 |
|  | #2 | “bispecific*”[tiab] OR “bi-specific*”[tiab] OR “2specific*”[tiab] OR “2-specific*”[tiab] OR “two-specific*”[tiab] OR “double-specific*”[tiab] OR “double specific*”[tiab] OR “dualspecific*”[tiab] OR “dual-specific*”[tiab] OR “polyspecific*”[tiab] OR “poly-specific*”[tiab] OR “Antibodies, Bispecific”[MeSH] OR “dual-target*”[tiab] OR “dualtarget*”[tiab] OR “dual target*”[tiab] OR “dual-antibod*”[tiab] OR “dual-antibod*”[tiab] OR “dual antibod*”[tiab] OR “dualantibod*”[tiab] OR “BiTE”[tiab] OR “AMG”[tiab] OR “REGN*”[tiab] OR “*engager*”[tiab] | 58,184 |
|  | #3 | “Review”[Publication Type] OR “Review Literature as Topic”[MeSH] OR “Systematic Review”[Publication Type] OR “Systematic Reviews as Topic”[MeSH] OR “Meta-Analysis”[Publication Type] OR “Meta-Analysis as Topic”[MeSH] OR “Network Meta-Analysis”[MeSH] | 3,180,782 |
|  | #4 | #1 AND #2 NOT #3 | 547 |
| Scopus (6.10.2022) | | | |
|  | #1 | TITLE-ABS-KEY(“Multiple Myeloma*” OR “plasma cell dyscrasia” OR “plasma-cell dyscrasia” OR “Myelomatosis” OR “Myelomatoses” OR “Kahler Disease” OR “Multiple Myeloma” OR “Myeloma-Multiple*”) | 79,257 |
|  | #2 | TITLE-ABS-KEY(“bispecific*” OR “bi-specific*” OR “2specific*” OR “2-specific*” OR “two-specific*” OR “double-specific*” OR “double specific*” OR “dualspecific*” OR “dual-specific*” OR “polyspecific*” OR “poly-specific*” OR “Antibodies, Bispecific” OR “dual-target*” OR “dualtarget*” OR “dual target*” OR “dual-antibod*” OR “dual-antibod*” OR “dual antibod*” OR “dualantibod*” OR “BiTE” OR “AMG” OR “REGN” OR “engager*”) | 126,869 |
|  | #3 | TITLE-ABS-KEY(“Review” OR “Review of Literature” OR “Systematic Review” OR “Meta-Analysis” OR “Meta Analysis” OR “Network Meta-Analysis” OR “Network Meta Analysis”) | 5,652,566 |
|  | #4 | #1 AND #2 NOT #3 | 260 |
| Web of Science (6.10.2022) | | | |
|  | #1 | TS=(“Multiple Myeloma*” OR “plasma cell dyscrasia” OR “plasma-cell dyscrasia” OR “Myelomatosis” OR “Myelomatoses” OR “Kahler Disease” OR “Multiple Myeloma” OR “Myeloma-Multiple*”) | 67,539 |
|  | #2 | TS=(“bispecific*” OR “bi-specific*” OR “2specific*” OR “2-specific*” OR “two-specific*” OR “double-specific*” OR “double specific*” OR “dualspecific*” OR “dual-specific*” OR “polyspecific*” OR “poly-specific*” OR “Antibodies, Bispecific” OR “dual-target*” OR “dualtarget*” OR “dual target*” OR “dual-antibod*” OR “dual-antibod*” OR “dual antibod*” OR “dualantibod*” OR “BiTE” OR “AMG” OR “REGN” OR “engager*”) | 68,339 |
|  | #3 | TS=(“Review” OR “Review of Literature” OR “Systematic Review” OR “Meta-Analysis” OR “Meta Analysis” OR “Network Meta-Analysis” OR “Network Meta Analysis”) | 2,231,865 |
|  | #4 | #1 AND #2 NOT #3 | 341 |
| EMBASE (6.10.2022) | | | |
|  | #1 | (“Multiple Myeloma*” OR “plasma cell dyscrasia” OR “plasma-cell dyscrasia” OR “Myelomatosis” OR “Myelomatoses” OR “Kahler Disease” OR “Multiple Myeloma” OR “Myeloma-Multiple*”):ab,ti | 79,309 |
|  | #2 | (“bispecific*” OR “bi-specific*” OR “2specific*” OR “2-specific*” OR “two-specific*” OR “double-specific*” OR “double specific*” OR “dualspecific*” OR “dual-specific*” OR “polyspecific*” OR “poly-specific*” OR “Antibodies, Bispecific” OR “dual-target*” OR “dualtarget*” OR “dual target*” OR “dual-antibod*” OR “dual-antibod*” OR “dual antibod*” OR “dualantibod*” OR “BiTE” OR “AMG” OR “REGN” OR “engager*”):ab,ti | 75,685 |
|  | #3 | (“Review” OR “Review of Literature” OR “Systematic Review” OR “Meta-Analysis” OR “Meta Analysis” OR “Network Meta-Analysis” OR “Network Meta Analysis”):ab,ti | 2,591,763 |
|  | #4 | #1 AND #2 NOT #3 | 613 |

**Table S2.** MINORS scale for quality assessment of included studies

|  | A clearly stated aim | Inclusion of consecutive patients | Prospective collection of data | Endpoints appropriate to the aim of the study | Unbiased assessment of the study endpoint | Follow-up period appropriate to the study's aim | Loss to follow-up less than 5% | Prospective calculation of the study size | Total |
| --- | --- | --- | --- | --- | --- | --- | --- | --- | --- |
| AMG 420  (BI 836909) | 2 | 2 | 2 | 2 | 0 | 2 | 2 | 0 | 12 |
| AMG 701 | 2 | 1 | 2 | 2 | 0 | 1 | 2 | 0 | 10 |
| Cevostamab  (BFCR4350A/ RO7187797) | 2 | 1 | 2 | 2 | 0 | 2 | 2 | 0 | 11 |
| CC-93269 | 2 | 1 | 2 | 2 | 0 | 1 | 2 | 0 | 10 |
| REGN5458 | 2 | 1 | 2 | 2 | 0 | 1 | 2 | 0 | 10 |
| Elranatamab  (PF-06863135) | 2 | 1 | 2 | 2 | 0 | 2 | 2 | 0 | 11 |
| TNB-383B  (ABBV-383) | 2 | 1 | 2 | 2 | 0 | 2 | 2 | 0 | 11 |
| Teclistamab  (JNJ-64007957) | 2 | 2 | 2 | 2 | 0 | 2 | 2 | 0 | 12 |
| Talquetamab  (JNJ-64407564) | 2 | 1 | 2 | 2 | 0 | 2 | 2 | 0 | 11 |

**Table S3.** Baseline characteristics of included studies

| Bispecific antibody | Patient inclusion | Exclusion of patients with a history of CNS involvement by myeloma | Exclusion of Prior BCMA targeted therapy | ECOG performance status | ISS stage | Median time from disease diagnosis (Year) | Prior history of HSCT |
| --- | --- | --- | --- | --- | --- | --- | --- |
| AMG 420  (BI 836909) | 1. At least two prior treatment regimens, including both PI as well as an IMID. 2. ECOG Performance Status 0, 1 or 2. | Yes | N/A | 0=57 II=40 III=2 | N/A | 5.2 (range: 1.3-20.0) | 36 (autologous only, allogenic recipients were excluded) |
| AMG 701 | 1. Relapsed after at least 3 lines of prior therapy that must include at a minimum of PI, an IMiD, and a CD38-directed cytolytic antibody  (Subjects who could not tolerate a PI, IMiDs, or a CD38-directed cytolytic antibody are eligible to enroll in the study).  2. ECOG Performance Status of ≤2. | Yes | Yes | N/A | I=18  II=41  III=22 | 5.6 (range: 0.5-15.1) | 9 allogenic, 68 autologous |
| Cevostamab  (BFCR4350A/ RO7187797) | 1. Patients with R/R MM for which there is no available, appropriate, or tolerable treatment.  2. ECOG Performance Status of ≤1. | Yes | No | N/A | N/A | 6.1 (range: 0.3-22.8) | N/A |
| CC-93269 | 1. Patients with R/R MM after ≥ 3 regimens; progressive disease within 60 days of last regimen.  2. ECOG Performance Status of ≤1. | Yes | Yes | 0=8  1=22 | I=9  II=11  III=9 | 5.9 (range: 1.4-16.6) | 3 allogenic, 23 autologous |
| REGN5458 | 1. Patients with MM who are R/R to ≥3 lines of prior therapy including an IMiD, a PI, and an anti-CD38 Ab, or double refractory to an IMiD and PI with PD on/after anti-CD38 Ab.  2. ECOG Performance Status of ≤2. | Yes | N/A | 0=21  1=51  2=1 | I=11  II=42  III=17 | N/A | 47 autologous |
| Elranatamab  (PF-06863135) | 1. Patients progressed or are intolerant of established therapies including a PI, an IMID, and an anti-CD38 Ab.  2. ECOG Performance Status of 0- 1 ( Performance Score 2 is permitted only if due to underlying myeloma). | N/A | No | N/A | I=6  II=12  III=7 | N/A | N/A |
| TNB-383B  (ABBV-383) | 1. Patients with R/R MM (i.e. ≥3 prior lines of therapy that included a PI, an IMID, and an anti-CD38 monoclonal antibody)  2. ECOG Performance Status of ≤2. | Yes | Yes | 0=38  1=67  2=11 | I=16  II=21  III=36 | N/A | 92 autologous |
| Teclistamab  (JNJ-64007957) | 1. Prior lines of therapy must include a PI, an IMID and anti-CD38 monoclonal antibody in any order during the course of treatment.  2. ECOG Performance Status score of 0 or 1. | Yes | Yes | 0=61  1=95 | I=75  II=49  III=32 | 6.6 (IQR: 3.9-10.1) | 133 |
| Talquetamab  (JNJ-64407564) | 1. Patients with R/R MM that are intolerant to established anti-MM treatments.  2. ECOG Performance Status score of 0 or 1. | Yes | No | N/A | N/A | 7 (range: 1-27) | 135 |

**Abbreviations**: IMiD: Immunomodulatory imide drug; PI: Proteasome inhibitors; MM: Multiple myeloma; R/R: Relapsed/refractory; ECOG: Eastern cooperative oncology group; BCMA: B-cell maturation antigen; ISS: International staging system; HSCT: Hematopoietic stem cell transplantation; N/A: Not available.

**Table S4.** Efficacy parameters for different bispecific antibodies

| BsAb | Dose | Total number | ORR | sCR/CR | VGPR | PR | MRD negativity | Median DOR (months) | Median time to first confirmed response |
| --- | --- | --- | --- | --- | --- | --- | --- | --- | --- |
| AMG 420 | All | 42 | 31% | 21% | 5% | 5% | 6/9 MRD negativity at <10^-4^ in patients with CR.  MRD-negative responses lasted for a median of 9.6 months (range: 2.8 to 12.8 months). | 8.4 (range: 2.5-15.5) | 1 months |
|  | 400 µg/d | 10 | 70% | 50% | 10% | 10% |  |  |  |
| AMG 701 | All | 82 | 26% | 10% | 7% | 9% | 6/7 MRD negativity at <10^-5^ in patients with ≥ VGPR.  All 6 patients have ongoing responses; 22-month MRD-negative response in 1 patient. | NR | N/A |
|  | 0.015-1.6 mg | 27 | 4% | 0 | 0 | 4% |  |  |  |
|  | 3-18 mg | 55 | 36% | 14% | 11% | 11% |  |  |  |
|  | 9 mg (Most recent evaluable cohort) | 6 | 83% | 17% | 33% | 33% |  |  |  |
| Cevostamab  (BFCR4350A/ RO7187797) | All | 161 | 40% | 8% | 15% | 17% | 7/10 MRD negativity at <10^-5^ in patients with ≥VGPR. | 11.5 (95% CI: 6.0-18.4) | 1 (range: 0.7-5.9) |
|  | 132-198 mg | 60 | 56% | 8% | 25% | 23% |  |  |  |
|  | 20-90 mg | 83 | 36% | 10% | 11% | 16% |  |  |  |
| CC-93269 | All | 30 | 43% | 17% | 13% | 13% | 12/13 MRD negativity at <10^-5^ in responding patients. | NR | 4.1 week (range: 4-13.1) |
|  | 3 → 6 mg and 6 mg | 14 | 35% | 7% | 7% | 21% |  |  |  |
|  | 6 → 10 mg and 10 mg | 9 | 89% | 45% | 33% | 11% |  |  |  |
| REGN5458 | All | 69 | 51% | 22% | 22% | 7% | 4/10 MRD negativity at <10^-5^ in patients with sCR/CR. | NR | <1 month |
|  | 3-12 mg | 24 | 29% | 26% | 0% | 3% |  |  |  |
|  | 24-96 mg | 25 | 48% | 24% | 24% | 0% |  |  |  |
|  | 200-800 mg | 24 | 75% | 16% | 42% | 17% |  |  |  |
| Elranatamab  (PF-06863135) | All | 30 | 46% | 20% | 23% | 3% | 3/3 patients assessed were MRD negative. | NR | 22 days (range: 21-50) |
|  | ≥215 µg/kg | 20 | 70% | 30% | 35% | 5% |  |  |  |
|  | 1000 µg/kg | 6 | 84% | 17% | 50% | 17% |  |  |  |
| TNB-383B  (ABBV-383) | ≥40 mg | 26 | 81% | 39% | 31% | 12% | N/A | NR | N/A |
|  | ≥40 mg + 60 mg dose expansion | 60 | 60% | 20% | 20% | 20% |  |  |  |
| Teclistamab  (JNJ-64007957) | SC | 72 | 64% | 39% | 21% | 4% | 44/54 MRD negativity at <10^-5^ in patients with sCR/CR. | 18.4 (95% CI: 14.9-NR) | 1.2 month (range: 0.2-5.5) |
|  | IV | 83 | 40% | 22% | 14% | 4% |  |  |  |
|  | 1.5 mg/kg SC | 165 | 63% | 39% | 19% | 4% |  |  |  |
| Talquetamab  (JNJ-64407564) | 405 μg/kg SC | 30 | 70% | 30% | 26.7% | 13.3% | N/A | 10.2 (range: 3.0-NR)  13.0 (range: 5.3-NR) | 0.9 month (range: 0.2-3.8)  1.2 month (range: 0.3-6.8) |
|  | 800 μg/kg SC | 44 | 63.6% | 20.5% | 36.4% | 6.8% |  |  |  |

**Abbreviations**: ORR: Objective response rate; sCR: Stringent complete response; VGPR: Very good partial response; PR: Partial response; MRD: Minimal residual disease; DOR: Duration of response; SC: Subcutaneous; IV: Intravenous; NR: Not reached; N/A: Not available.





**Figure S1.** All hematologic adverse events following administration of bispecific antibodies for patients with multiple myeloma. A) Anemia, B) Neutropenia, C) Thrombocytopenia, D) Leukopenia, and E) Lymphopenia.





**Figure S2.** All non-hematologic adverse events following administration of bispecific antibodies for patients with multiple myeloma. A) Infection, B) Dysgeusia, C) Fatigue, D) Diarrhea, E) Nausea, F) Pyrexia, G) Headache, H) Cough, I) Back pain, J) Vomiting, K) AST increase, L) ICANS, M) Neurotoxicity, and N) Death. Abbreviations: AST: Aspartate Transferase; and ICANS: Immune effector cell-associated neurotoxicity syndrome.





**Figure S3.** Grade ≥3 hematologic adverse events following administration of bispecific antibodies for patients with multiple myeloma. A) Neutropenia, B) Anemia, C) Lymphopenia, D) Thrombocytopenia, and E) Leukopenia.





**Figure S4.** Grade ≥3 non-hematologic adverse events following administration of bispecific antibodies for patients with multiple myeloma. A) AST increase, B) Back pain, C) Diarrhea, D) Fatigue, E) Headache, F) Cough, G) Nausea, H) Vomiting, and I) Pyrexia. Abbreviations: AST: Aspartate Transferase.


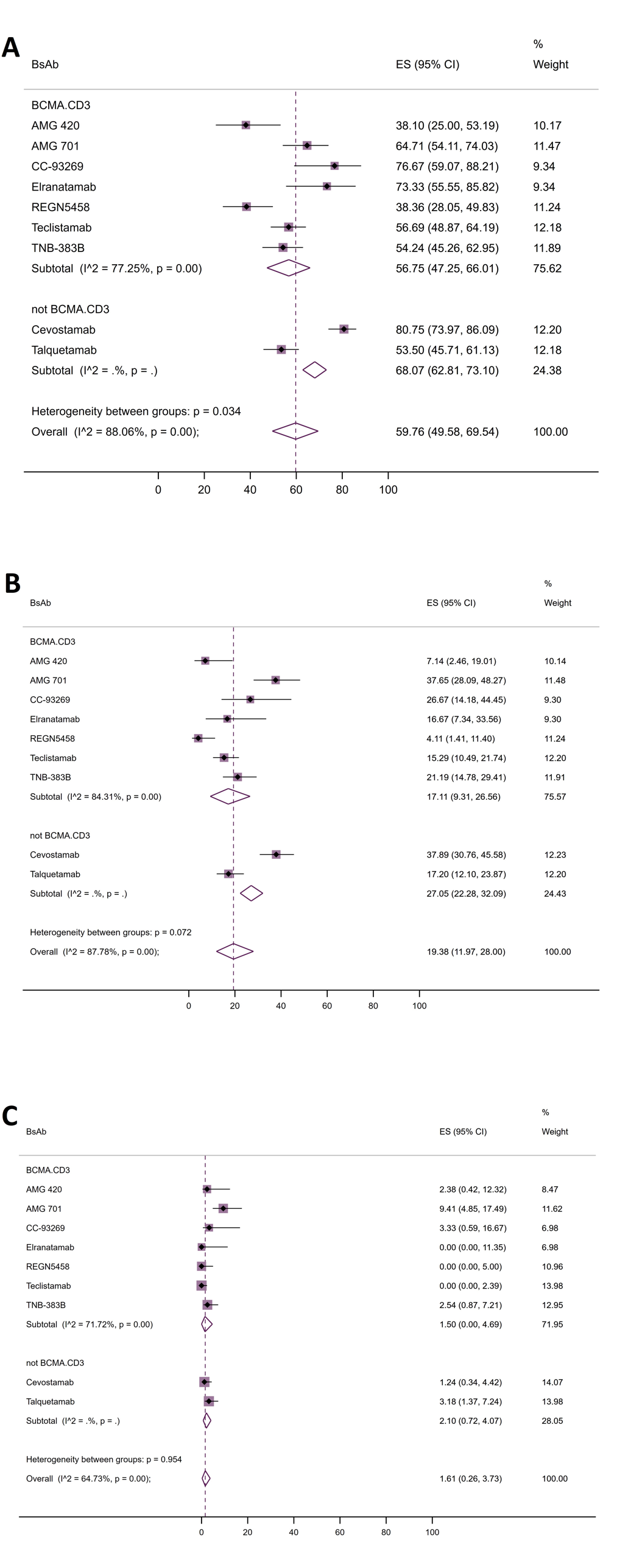


**Figure S5.** ‎Cytokine release syndrome (CRS) events following administration of bispecific antibodies for patients with multiple myeloma. A) All events, B) CRS events grade ≥2, and C) CRS events grade ≥3
